# Transcriptional landscape of intestinal environment in DSS-induced ulcerative colitis mouse model

**DOI:** 10.1186/s12876-024-03128-8

**Published:** 2024-02-02

**Authors:** Yuefei Wen, Chenyang Li, Peng Huang, Zhigang Liu, Yanjun He, Bin Liu

**Affiliations:** 1 Foshan Maternity and Child Health Care Hospital, Southern Medical University, 528000 Foshan, China; 2 Zhujiang Hospital, Southern Medical University, 510282 Guangzhou, China

**Keywords:** Ulcerative colitis, RNA-seq, Pathway enrichment analysis, Protein-protein interaction (PPI) network, ceRNA network analysis

## Abstract

Ulcerative colitis (UC) is a chronic inflammatory disease that targets the colon and has seen an increasing prevalence worldwide. In our pursuit of new diagnostic and therapeutic approaches for UC, we undertook a sequencing of colons from UC mouse models. We focused on analyzing their differentially expressed genes (DEGs), enriching pathways, and constructing protein-protein interaction (PPI) and Competing Endogenous RNA (ceRNA) networks. Our analysis highlighted novel DEGs such as Tppp3, Saa3, Cemip, Pappa, and Nr1d1. These DEGs predominantly play roles in pathways like cytokine-mediated signaling, extracellular matrix organization, extracellular structure organization, and external encapsulating structure organization. This suggests that the UC pathogenesis is intricately linked to the interactions between immune and non-immune cells with the extracellular matrix (ECM). To corroborate our findings, we also verified certain DEGs through quantitative real-time PCR. Within the PPI network, nodes like Stat3, Il1b, Mmp3, and Lgals3 emerged as significant and were identified to be involved in the crucial cytokine-mediated signaling pathway, which is central to inflammation. Our ceRNA network analysis further brought to light the role of the Smad7 Long non-coding RNA (lncRNA). Key MicroRNA (miRNAs) in the ceRNA network were pinpointed as mmu-miR-17-5p, mmu-miR-93-5p, mmu-miR-20b-5p, mmu-miR-16-5p, and mmu-miR-106a-5p, while central mRNAs included Egln3, Plagl2, Sema7a, Arrdc3, and Stat3. These insights imply that ceRNA networks are influential in UC progression and could provide further clarity on its pathogenesis.

In conclusion, this research deepens our understanding of UC pathogenesis and paves the way for potential new diagnostic and therapeutic methods. Nevertheless, to solidify our findings, additional experiments are essential to confirm the roles and molecular interplay of the identified DEGs in UC.

**Supplementary Information:**

The online version contains supplementary material available at 10.1186/s12876-024-03128-8.

## Introduction

Ulcerative colitis (UC) is a chronic inflammatory bowel disease that predominantly affects the colon. Originating from multiple sources, its complexities lie in factors such as genetic predispositions, anomalies in the epithelial barrier, misaligned immune responses, and environmental elements [1].

UC’s incidence is steadily increasing worldwide [2]. The figures are concerning, with incidences ranging from 9 to 20 cases per 100,000 person-years, and prevalence oscillating between 156 to 291 cases per 100,000 people [1]. The symptoms of UC can be severe and have a significant impact on the quality of life of affected individuals [3, 4]. Moreover, studies link UC to subsequent psychological challenges, further adding to the burden of the disease [5–7].

One alarming consequence of UC is its potential progression to colorectal cancer (CRC) [8]. For those living with UC over three decades, the odds of developing colon cancer can rise up to 20% [9, 10]. UC-induced CRC patients often tend to be of a younger demographic and exhibit a propensity for multiple malignant lesions, and histologically exhibit mucinous or signet ring cell carcinomas [11].

Both UC and UC-related cancer are believed to be intrinsically linked to sustained inflammation in the colon [12]. Although the definitive etiology of inflammatory bowel disease (IBD) is yet to be pinpointed, it’s speculated to be a nexus of genetic, environmental, microbial, and immune factors [13]. Existing medical interventions for IBD primarily aim at curtailing the mucosal inflammation to alleviate associated symptoms. Nevertheless, for a segment of patients, the treatment journey extends to encompassing cancer therapies like chemotherapy, radiation, or surgical procedures [14, 15].

Our present research is anchored in unearthing the underlying intricacies of UC pathogenesis. By leveraging UC mouse models, we probed its DEGs. Comprehensive functional assessments were undertaken, encompassing enrichment analysis, protein-protein interactions, the Competing Endogenous RNA (ceRNA) network, and relevant pathway enrichments. These endeavors cast light on the multifaceted molecular mechanisms integral to UC.

## Materials and methods

### Animal experimental protocol

The National Institutional Animal Care and Ethical Committee of Southern Medical University authorized all animal care procedures. All animal testing was conducted in accordance with the Guide for the Care and Use of Laboratory Animals. C57BL/6 mice used in the experiment were purchased from the Southern Medical University Laboratory Animal Center, China. Eight-week-old male C57BL/6J mice were housed under controlled conditions of $25^{\circ }$C temperature, 45-55% humidity, and 12 h light/dark cycle.

Six mice were randomized into two groups: those receiving Dextran sodium sulphate (DSS) (UC group) and those receiving a placebo (control group). To induce UC models in the experiments, 3% DSS (D122347, Aladdin, Shanghai, China) was dissolved in drinking water for seven days [16]. The mice in the control group were only given water.

After injecting the mice with Pentobarbital sodium, the ventral face was sprayed with 70% ethanol and a midline incision was made to delicately expose the peritoneal cavity. The colon was then removed by severing it just after the ileocecal junction and at the rectum’s terminus. Manual displacement with forceps and flushing with ice-cold phosphate buffered saline (PBS) using a blunt needle affixed to a syringe were used to remove feces with care. Colon tissue was cut for RNA-seq analysis [16]. Finally, we performed terminal bleeding and euthanized mice in accordance with approved institutional animal ethical protocols.

### RNA sequencing

TRIzol (Invitrogen, Carlsbad, CA, USA) was utilized to isolate RNA from mouse colon tissue utilizing multiplex PCR amplification techniques. According to Miyuraj et al. [17], using Oligo (dT)25 magnetic beads and deoxy-ribonucleoside triphosphate (dNTP) Mix etc., mRNA sequencing libraries were constructed. mRNA sequencing was performed on the Illumina sequencing platform NextSeq 550, while microRNA sequencing was performed on the Illumina sequencing platform HiSeq 4000.

### Total RNA extraction and quantitative real-time PCR

Total RNA was extracted from brain or cell samples using the TRIzol reagent (Invitrogen, Carlsbad, CA, USA). RNA (1 $\upmu$g) was reverse transcribed to cDNA using a Hifair® II First-strand cDNA Synthesis Kit (Yeasen Biotech, Shanghai, China). mRNA expression levels were quantified using a SYBR Green Master Mix (Exiqon, Vedbaek, Denmark), and Ct values for each sample and gene were normalized with respect to glyceraldehyde 3-phosphate dehydrogenase. The expression of miRNA was tested using a fast real-time PCR system (7900 HT, ABI, Foster City, CA) and the appropriate miRNA oligonucleotide primers (Qiagen, Hilden, Germany). The fold-change values were calculated by normalizing with respect to the control samples. PCR amplification was performed for 40 cycles, and the data were collected using SDS software (Applied Biosystems, Foster City, CA). The sequences of the mRNA oligonucleotide primers were used are listed in Table 1.

**Table 1:** The sequences of the mRNA oligonucleotide primers

Gene	Forward Sequences	Reverse Sequences
Mouse Tppp3	AGCGGGCAAGAGATGAATGG	GCAGATTTCGCCTTGACTTTG
Mouse Saa3	TGCCATCATTCTTTGCATCTTGA	CCGTGAACTTCTGAACAGCCT
Mouse Nr1d1	TACATTGGCTCTAGTGGCTCC	CAGTAGGTGATGGTGGGAAGTA
Mouse Gapdh	AGGTCGGTGTGAACGGATTTG	TGTAGACCATGTAGTTGAGGTCA

### Mapping

We used FastQC and Trimmomatic [18] to remove adaptors. mRNA was aligned using STAR software [19] and the reference sequence mm10, while miRNA (microRNA) was aligned using miRBase data. Using R software, downstream statistical analyses were conducted.

### Differential expression analysis

The differential analysis of mRNA expression was performed using DESeq2 [20]. To highlight the top genes, the EnhancedVolcano package was used to generate volcano plots with a default cutoff for log2FC $>|2|$ and a default cutoff for a *p*-value of 10e-6.

### Differential miRNA: mRNA interactions

multiMiR was used to search multiple miRNA-mRNA databases for miRNAs [21]. Using a binomial test, the differential miRNA-mRNA interaction was calculated. Additionally, False Discovery Rate (FDR) was used to adjust for multiple tests.

### ceRNA network analysis

lncRNA2Target [22] was used to search for potential lncRNAs (long noncoding RNAs) targeting DEGs for the analysis of ceRNAs. Additionally, the ceRNA network of the collected miRNAs and lncRNAs was built and visualized using the igraph package by querying interactions between them from multiple miRNA-lncRNA databases from multiMiR. The ceRNAs were also used to perform pathway enrichment.

### Protein-protein interaction network analysis

The protein-protein interaction (PPI) network of the mRNA DEGs was analyzed with the R package STRINGdb [23] to generate an interaction table, and the interaction network was visualized with the igraph package.

## Results

### Identification of differentially expressed genes

The DEG analysis identified disparities in gene expression between the UC and control groups (see Additional file 1).

**Fig. 1:**
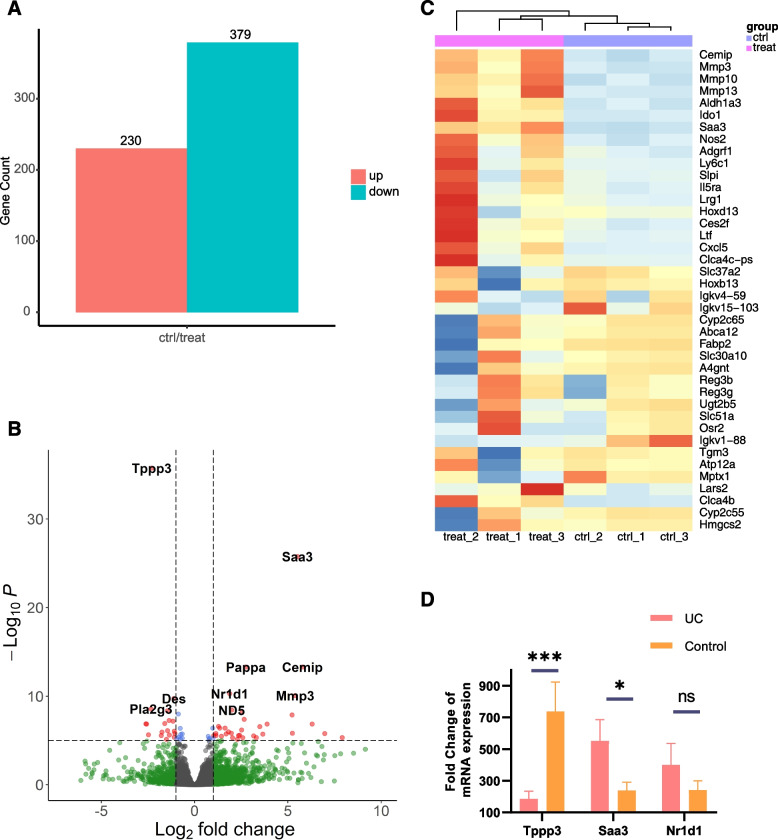
Separated DEGs into two categories. **A** A bar graph displaying DEG statistical data. **B** A volcano plot of DEGs between the UC group and the control group. **C** Clustering of samples based on the expression level of the leading DEGs. Blue and green scatter points represent insignificant DEGs in the Volcano plot, while red scatter points represent upregulated DEGs and blue scatter points represent downregulated DEGs. The statistical method uses the default cutoff for log2FC, which is $>|2|$, and the default cutoff for *p*-value, which is 10e-6, to highlight the most significant genes in red. **D** Tppp3, Saa3, Nr1d1, and Pappa expression in the controls and UC groups 7 days after DSS (*n* = 6). Data are presented as mean ± SEM. *p < 0.05, **p < 0.01, ***p < 0.001

Our gene expression study detected notable changes in 379 upregulated genes and 230 downregulated genes in UC samples compared to controls (Fig. 1A). The accompanying volcano plot accentuated the primary DEGs, which notably encompassed Tppp3, Saa3, Cemip, Pappa, and Nr1d1, all with a significance level of p<0.001 (Fig. 1B). A heatmap integrated with sample clustering analysis manifested that the majority of the upregulated genes were in the top 50 genes in response to UC groups (Fig. 1C).

Delving deeper into the foremost five DEGs, Tppp3 showcased significantly enhanced expression in controls relative to UC groups ($log_{2}FC=-2.30$) ($log_{2}FoldChange$). In contrast, the expression levels of Saa3 ($log_{2}FC=5.52$), Cemip ($log_{2}FC=5.78$), Pappa ($log_{2}FC=2.74$), and Nr1d1 ($log_{2}FC=1.88$) were markedly escalated in the UC samples. Within this cluster of DEGs, Saa3 has been characterized to induce pathogenic Th17 cells, fostering inflammation [24]. Simultaneously, Nr1d1, intertwined with circadian rhythm modulation, and its altered expression has been associated with IBD [25].

Cemip, which is another DEG spotlighted in our study, has been implicated in promoting tumorigenic and metastatic activities. Its repertoire includes stimulating migration and invasion, inhibiting cell death, promoting survival, degrading hyaluronic acid(HA), regulating pro-metastatic signaling pathways, inducing the epithelial-mesenchymal transition(EMT) program, and steering metabolic reprogramming and premetastatic conditioning of future metastatic microenvironments [26].

In order to verify the DEGs, we also performed quantitative real-time PCR on colons tissues induced by DSS, and found that Tppp3 was highly expressed in controls, and Saa3 was indeed highly expressed in UC group(Fig. 1D). Nr1d1 did not show significant differences, possibly due to the small sample size.

### Pathway enrichment between groups

**Fig. 2:**
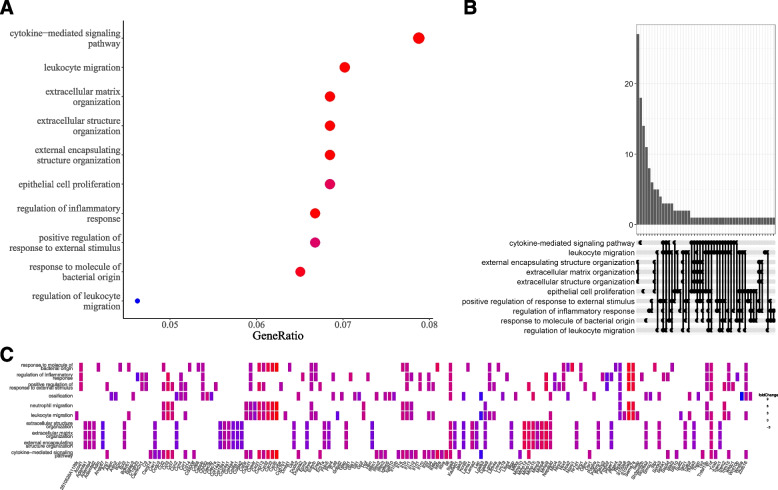
DEG enrichment in pathways between UCs and controls. **A** The top 10 enriched pathways in terms of GO for both the UC and control groups. **B** The top 10 enriched pathways for GO terms in the Upset plot for UC and control groups. **C** Heatmap of DEGs present in enriched pathways

The pathway enrichment analysis for DEGs unveiled notable distinctions in pathway activation when comparing UC mouse models to control groups. Through gene ontology (GO) term enrichment analysis, 1494 pathways were discerned (see Additional file 2). These encompassed pivotal pathways implicated in inflammation and tissue remodeling, such as cytokine-mediated signaling pathway, extracellular matrix organization, extracellular structure organization, and external encapsulating structure organization (Fig. 2A).

To delve deeper into the interconnections among these enriched pathways, a heatmap (Fig. 2B) and an UpSet plot (Fig. 2C) were formulated. The ensuing data pinpointed a subset of genes recurrently shared across multiple pathways, predominantly clustering within extracellular-associated pathways. This gene cluster included Mmp3, Mmp12, Col14a1, Mmp10, and Ecm2. Significantly, each of these genes is intricately involved in extracellular matrix organization, extracellular structure organization, and external encapsulating structure organization.

The extracellular matrix (ECM) assumes a pivotal role in IBD pathogenesis, serving as a foundational scaffold for cellular and tissue architecture and influencing the interplay between immune and non-immune cells [27]. Anomalies in ECM dynamics, including its remodeling and degradation, are believed to foster the onset and escalation of UC. Hence, the present insights underscore the potential therapeutic leverage in targeting genes associated with extracellular pathways for more effective UC intervention.

### Network analysis of the protein-protein interaction

**Fig. 3:**
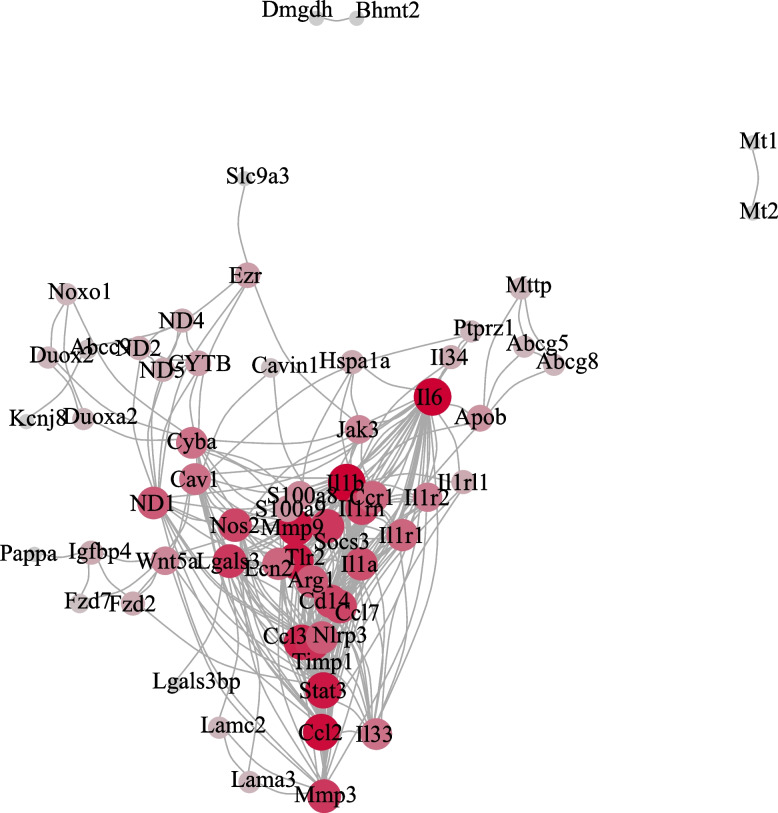
The outcome of the PPI network of the leading 61 DEGs in the UC and control groups

To understand the interactions between the DEGs and other molecules better, we performed a protein-protein interaction (PPI) analysis. This analysis yielded a PPI network derived from the DEGs, showcasing highly confident interactions. Such interactions suggest these proteins interplay in the treated mice. The network encompassed 61 nodes with a score higher than 982 and was supported by 302 interconnecting links. Notably, key hub genes emerged from these nodes, namely Stat3, Il1b, Mmp3, and Lgals3 (Fig. 3). These genes play pivotal roles in ulcerative colitis pathogenesis.

Specifically, Stat3 stands out as a central mediator of pathogenic gene transcription in IBD [28]. It is known to play a critical role in regulating immune responses and inflammation and promoting cell proliferation and survival. Within the context of UC, aberrant activation of Stat3 correlates with the release of proinflammatory cytokines and chemokines and the suppression of anti-inflammatory responses [29]. Such insights underline the imperative of focusing on Stat3 and similar hub genes when crafting new therapeutic strategies for UC.

### Network analysis of lncRNA-miRNA-mRNA ceRNAs

**Fig. 4:**
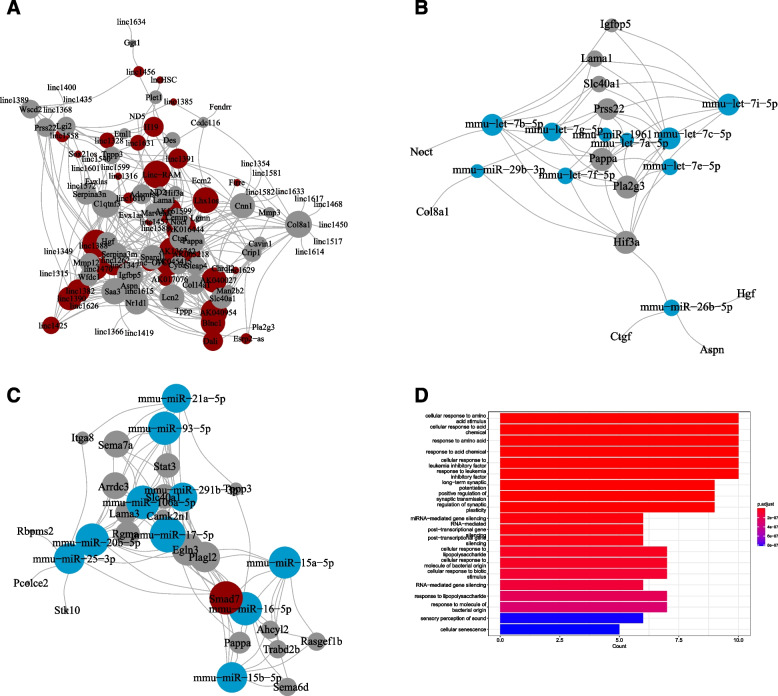
Result of a network analysis of ceRNAs. **A** lncRNA-mRNA network of all differentially expressed genes (DEGs) in UC and control groups. **B** miRNA-mRNA network of all DEGs in the UC and control groups. **C** the ceRNA network of all DEGs in UC and the control group. The vertex’s size indicates the number of connections. **D** The top 10 enriched pathways in terms of GO for ceRNAs

Utilizing lncRNA2Target, we extracted lncRNA-mRNA interaction data for both treatment and control groups. This analysis identified 342 interactions that included 46 DEGs and 63 lncRNAs, as presented in Fig. 4A. Among these DEGs, C1qtnf3, Col8a1, and Saa3 were prominent, interacting with most lncRNAs, while AK016444, AK045415, AK136742, and linc1388 targeted the majority of mRNAs.

In the miRNA-mRNA interaction, the hub genes for miRNA included mmu-let-7e-5p, mmu-let-7c-5p, and mmu-let-7b-5p. On the mRNA front, Pappa, Pla2g3, and Prss22 emerged as the central hubs, as illustrated in Fig. 4B.

A subsequent ceRNA network was reconstructed, highlighting the lncRNA Smad7. The hub miRNAs within this network were mmu-miR-17-5p, mmu-miR-93-5p, mmu-miR-20b-5p, mmu-miR-16-5p, and mmu-miR-106a-5p. Meanwhile, Egln3, Plagl2, Sema7a, Arrdc3, and Stat3 were identified as the pivotal (Fig. 4C).

It is noteworthy that Sema7a has been recognized for its role in stimulating colon macrophages to produce IL-10, aligning with previous research which attributes anti-colitis effects to Sema7A [30, 31].

Finally, ceRNA enrichment analysis indicated that these miRNAs have strong correlations with various cellular responses, including amino acid stimulus, acid chemical response, and leukemia inhibitory factor response (Fig. 4D). Such associations could hint at their role in the colon’s inflammation process.

## Discussion

UC is an increasingly prevalent chronic inflammatory disease of the colon globally. Its etiology is multifaceted, resulting from a sophisticated interplay between genetic predisposition, environmental influencers, altered immune responses, and abnormal epithelial barrier function [13]. The understanding of the genetic underpinnings of inflammatory bowel disease (IBD) has been significantly enhanced over the past few decades [32], thanks to technological advances in DNA analysis and sequencing, as well as the use of large-scale multinational databases [33].

The DSS-induced colitis model stands validated as an effective tool for assessing potential therapeutic compounds pertinent to human conditions [34]. This model’s histopathological markers are close to those observed in human IBDs [35], particularly UC. Importantly, the induced inflammatory environment encompasses features of both Crohn’s disease (CD) and UC [36].

In contrasting the UC and control groups, our DEG analysis identified pronounced disparities in gene expression. Specifically, within the UC group, 379 genes manifested heightened activity, while 230 genes displayed diminished activity compared to the control group. Notably, Tppp3 registered higher levels in the control group, whereas Saa3, Cemip, Pappa, and Nr1d1 were accentuated within the UC group. Both Saa3 and Nr1d1 established links to inflammatory events and IBD. To substantiate these DEG findings, we also implemented quantitative real-time PCR assessments.

Our pathway enrichment investigation pinpointed several crucial pathways involved in inflammation and tissue restructuring. These include inflammation and tissue remodeling, including cytokine-mediated signaling, extracellular matrix organization, and encapsulating structure organization. These pathways displayed marked activation within the UC group.

Protein-protein interaction analysis has underscored the significance of hub genes, notably Stat3, Il1b, Mmp3, and Lgals3, in the progression of ulcerative colitis. Among these, Stat3 stands out due to its association with immune response regulation, inflammation, and cell survival, highlighting its potential as a therapeutic focal point.

Furthermore, a network exploration of lncRNA-miRNA-mRNA ceRNA interactions has spotlighted several lncRNAs and miRNAs intricately connected with pivotal DEGs, such as C1qtnf3, Col8a1, Saa3, Pappa, Pla2g3, and Prss22. It’s worth emphasizing that Sema7a, a hub mRNA, plays a part in anti-colitis effects by stimulating macrophage IL-10 production.

Summarily, our observations provide a clearer picture of the molecular intricacies of ulcerative colitis and spotlight promising therapeutic avenues for addressing this specific inflammatory bowel disease. The delineated DEGs and pathways could potentially pave the way for innovative research and the development of breakthrough treatment protocols to better patient prognosis.

While our study offers valuable perspectives into the molecular dynamics governing UC, it is not without limitations. The employed mouse sample size is relatively constrained, which might temper the conclusiveness of our findings. Furthermore, given the intricate nature of gene functionalities, any predictions rooted solely in bioinformatics warrant corroboration through cellular and animal trials. However, the study’s contributions to elucidating genetic factors in UC hold promise for shaping future therapeutic interventions for this challenging condition.

## Conclusion

The current research illuminates the potential roles of DEGs and associated pathways in the onset and progression of UC when evaluating UC mouse models against controls. Delving into the ceRNA network-mediated genes, we unveiled probable molecular pathways underpinning UC’s pathogenesis.

Our findings offer new insights into the complex molecular processes that contribute to the onset and progression of UC. Leveraging advanced bioinformatics methodologies, we pinpointed pivotal DEGs and pathways likely instrumental in the trajectory of this chronic inflammatory ailment.

We anticipate that these findings will serve as a stepping stone for deeper exploration into UC’s underpinnings, and the formulation of innovative therapeutic approaches tailored for this debilitating disorder. By elucidating the molecular foundation of UC, our endeavor holds the potential to significantly enhance the living standards of countless individuals globally afflicted by this condition.

### Supplementary Information

**Additional file 1.** DEG analysis result.

**Additional file 2.** Gene ontology (GO) term enrichment analysis result.

**Additional file 3.** A doc file of 3 supplementary figures.

## Data Availability

The datasets for this study can be found in the NCBI repository PRJNA938800.

## Abbreviations

DSS: Dextran sodium sulphate
GO: Gene ontology
UC: Ulcerative colitis
PPI: Protein-protein interaction
DEG: Differentially expressed gene
CRC: Colorectal cancer
CD: Crohn’s disease
IBD: Inflammatory bowel disease
EMT: Epithelial-mesenchymal transition
HA: Hyaluronic acid
ECM: Extracellular matrix
FC: Fold change
FDR: False discovery rate
lncRNA: Long noncoding RNA
miRNA: microRNA
ceRNA: Competing endogenous RNA
PCR: Polymerase Chain Reaction
PBS: Phosphate buffered saline
