# Supplementary material for: Transcriptional landscape of intestinal environment in DSS-induced ulcerative colitis mouse model
Source: BMC Gastroenterol. 2024 Feb 2;24:60. doi: 10.1186/s12876-024-03128-8 (PMC10836045; doi:10.1186/s12876-024-03128-8)
Supplement: Supplementary file 3 — Additional file 3. A doc file of 3 supplementary figures. [file 12876_2024_3128_MOESM3_ESM.docx]

## Supplementary Figures


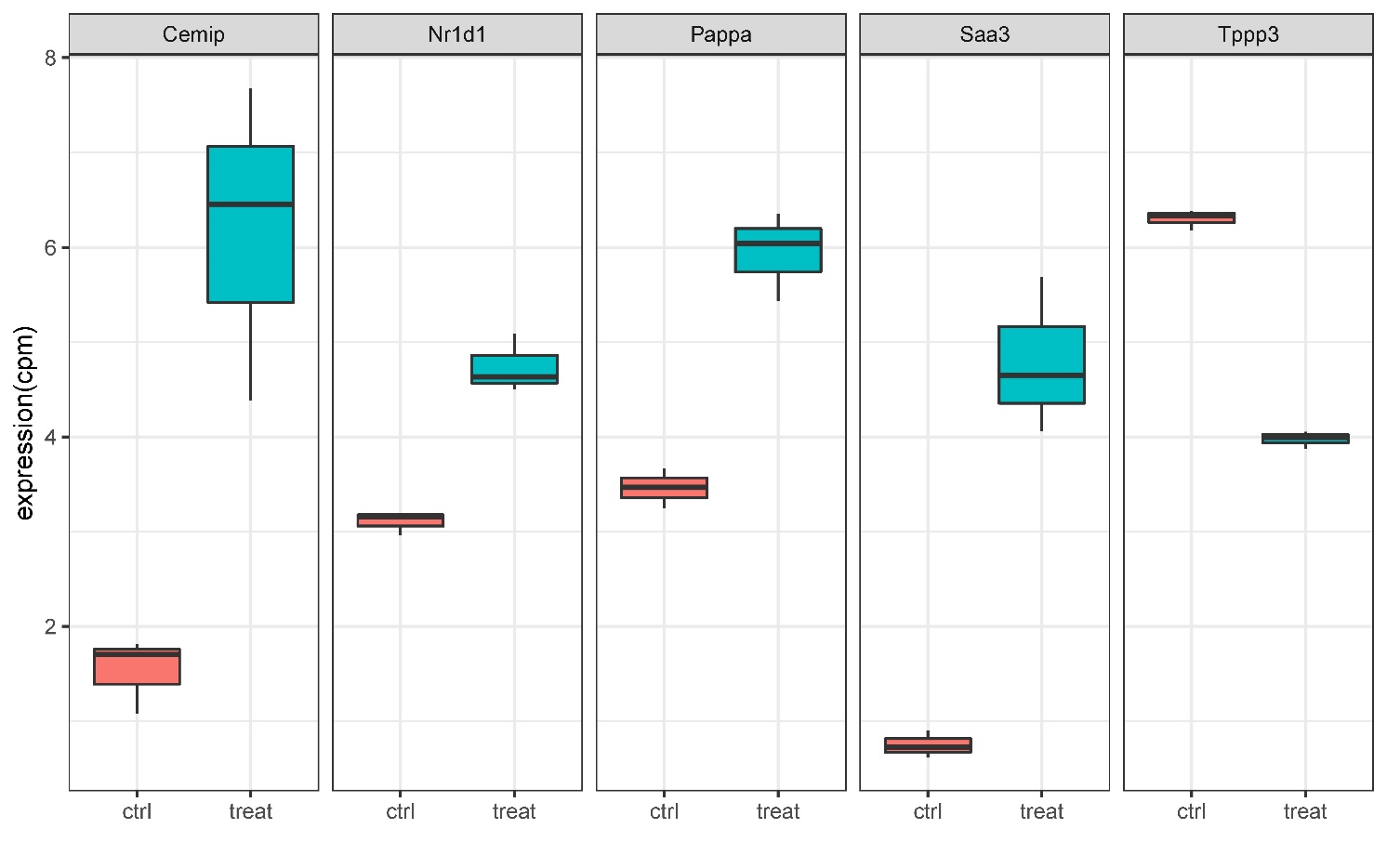


**Supplementary Figure 1.** DEGs result of UC vs Control. Boxplot of Top 5 differentially expressed mRNA.


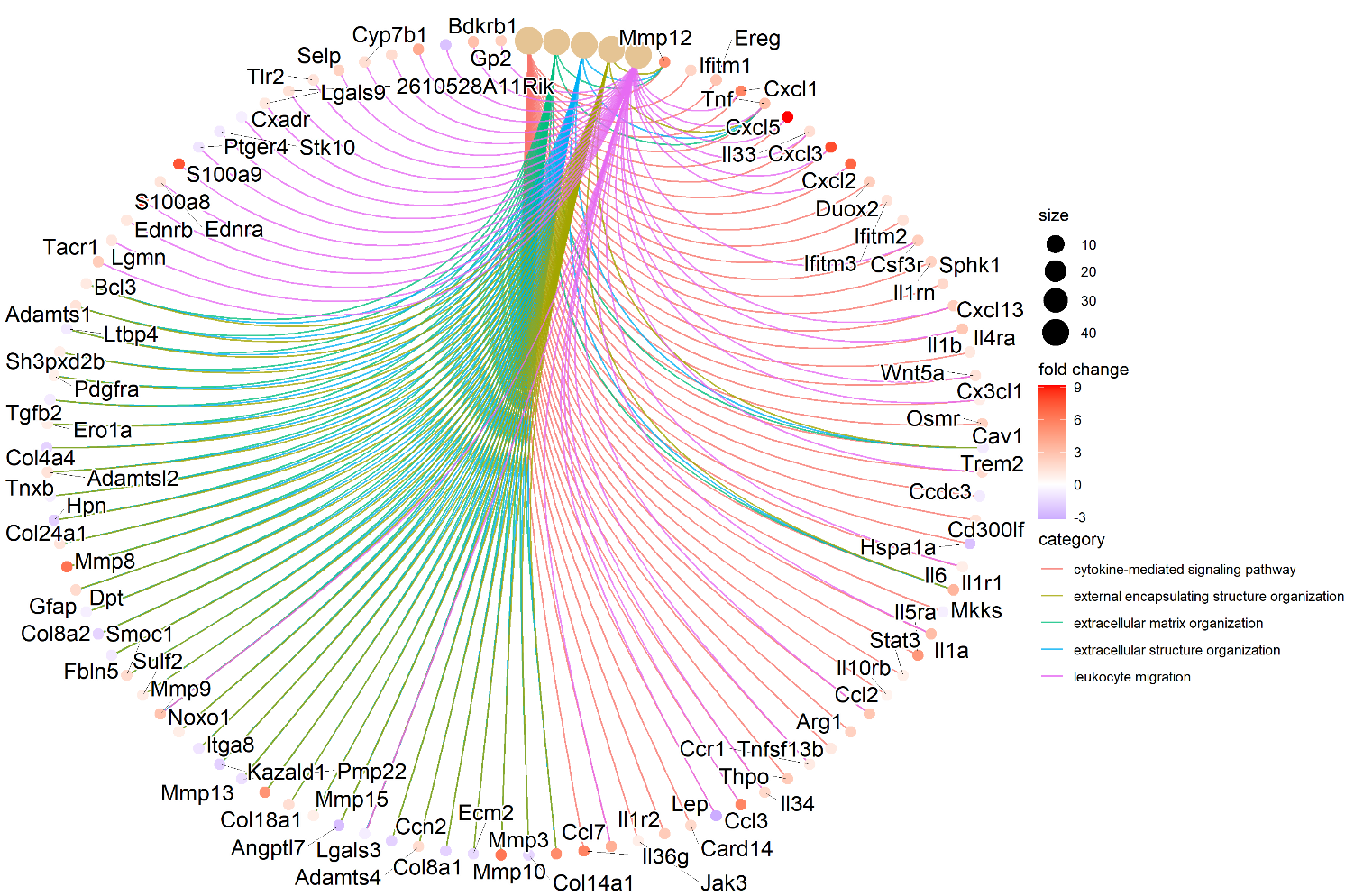


**Supplementary Figure 2.** CnetPlot shows pathways with relevant genes and overlap.


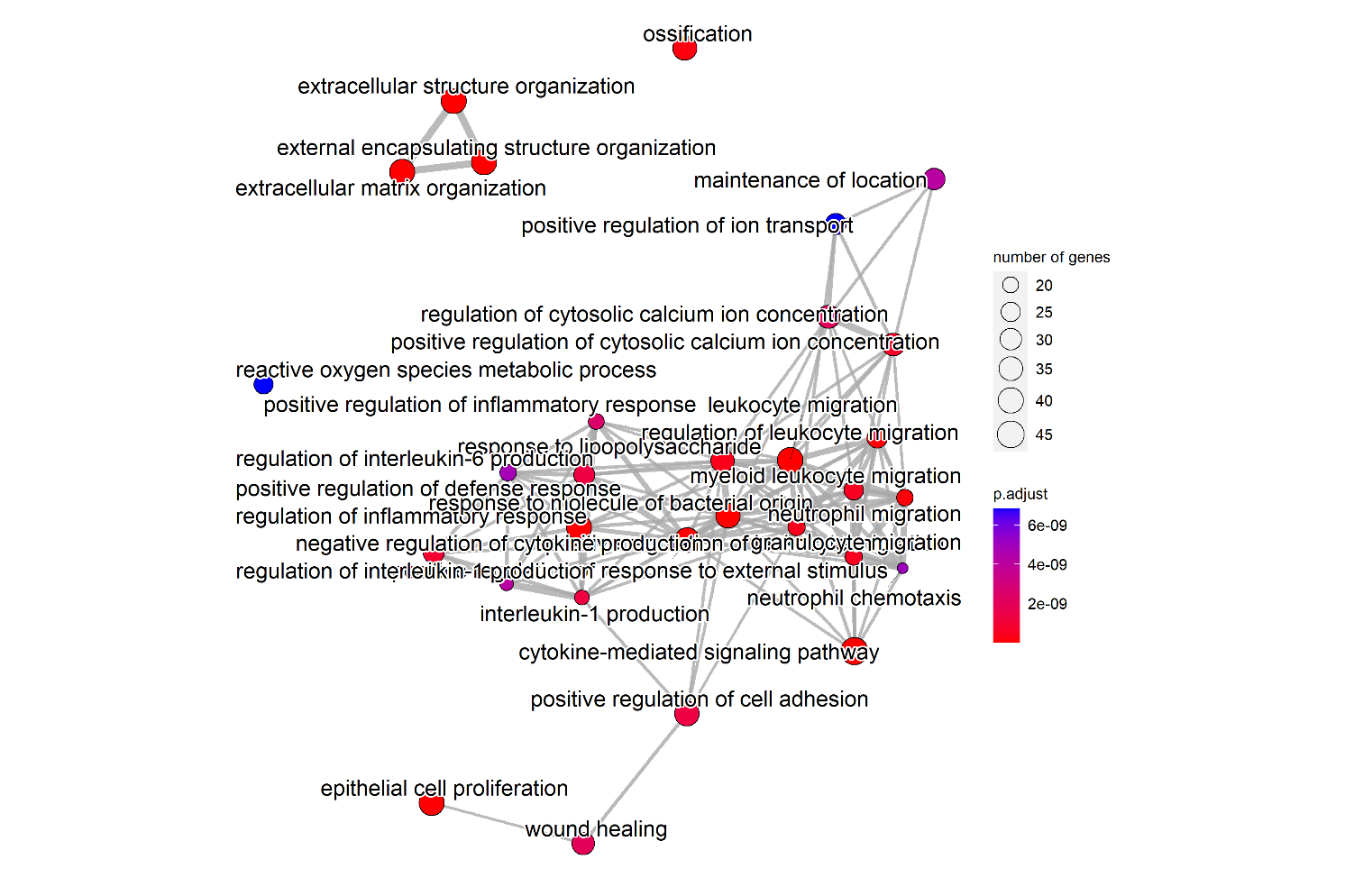


**Supplementary Figure 3.** Network plot of top 30 pathways.
